# The Profile and Antimicrobial Activity of *Bacillus* Lipopeptide Extracts of Five Potential Biocontrol Strains

**DOI:** 10.3389/fmicb.2017.00925

**Published:** 2017-05-23

**Authors:** Ivica Dimkić, Slaviša Stanković, Marija Nišavić, Marijana Petković, Petar Ristivojević, Djordje Fira, Tanja Berić

**Affiliations:** ^1^Department of Microbiology, Faculty of Biology, University of BelgradeBelgrade, Serbia; ^2^Department of Physical Chemistry, Institute of Nuclear Sciences “Vinča,” University of BelgradeBelgrade, Serbia; ^3^Innovation Centre of the Faculty of Chemistry Ltd., University of BelgradeBelgrade, Serbia; ^4^Department of Biochemistry and Molecular Biology, Faculty of Biology, University of BelgradeBelgrade, Serbia

**Keywords:** *Bacillus*, lipopeptides, MALDI-TOF MS, HPTLC, iturin, ethyl acetate extract, bioautography

## Abstract

In this study the efficacy of two different methods for extracting lipopeptides produced by five *Bacillus* strains-ethyl acetate extraction, and acid precipitation followed by methanol extraction—was investigated using mass spectrometry. High performance thin layer chromatography (HPTLC) was also used for the simultaneous separation of complex mixtures of lipopeptide extracts and for the determination of antimicrobial activity of their components. The mass spectra clearly showed well-resolved groups of peaks corresponding to different lipopeptide families (kurstakins, iturins, surfactins, and fengycins). The ethyl acetate extracts produced the most favorable results. The extracts of SS-12.6, SS-13.1, and SS-38.4 showed the highest inhibition zones. An iturin analog is responsible for the inhibition of *Xanthomonas arboricola* and *Pseudomonas syringae* phytopathogenic strains. HPTLC bioautography effectively identified the active compounds from a mixture of lipopeptide extracts, proving *in situ* its potential for use in direct detection and determination of antimicrobials. In the test of potential synergism among individual extracts used in different mixtures, stronger antimicrobial effects were not observed. Biochemical and phylogenetic analysis clustered isolates SS-12.6, SS-13.1, SS-27.2, and SS-38.4 together with *Bacillus amyloliquefaciens*, while SS-10.7 was more closely related to *Bacillus pumilus*.

## Introduction

Environmental contamination caused by the widespread use of pesticides and the appearance of multiple resistant strains has led to public awareness, and a demand for a reduction in the use of artificial chemical compounds. In that context, biological control through the use of natural microorganisms offers a promising alternative to industrial pesticides. The ability to produce various antimicrobial substances, and their sporulation capacity, give *Bacillus* strains a significant advantage in terms of their survival in different habitats. *Bacillus* species produce a wide array of biologically active compounds, including polyketides, lipopeptides, siderophores, and peptides (Stein, 2005; Ongena and Jacques, 2008; Hamdache et al., 2011). Many studies have shown that lipopeptides from the fengycin, surfactin, and iturin families have significant potential for fighting plant pathogens (Stein, 2005; Romero et al., 2007; Ongena and Jacques, 2008; Raaijmakers et al., 2010; Roongsawang et al., 2010; Romano et al., 2011; Dimkić et al., 2013, 2015). All lipopeptides contain a lipophilic fatty acid chain and a hydrophilic peptide ring (Toure et al., 2004). There are amphiphilic cyclic peptides comprised of 7 α-amino acids (surfactins and iturins), 10 α-amino acids (fengycins), those linked to a single β-amino fatty acid (iturins), and those linked to a β-hydroxy fatty acid (surfactins and fengycins). In addition to fengycin, surfactin and iturin, kurstakins represent a new family of lipopeptides. Although the first kurstakins to be isolated did not contain β-hydroxy fatty acid and were classified as linear molecules, it has been shown that they can be found in the form of partially cyclic compounds (Hathout et al., 2000), as well as in cyclic formations (Béchet et al., 2012), which puts them in a class of non-cationic cyclic lipopeptides (Cochrane and Vederas, 2016).

Detection of antibiotic production by particular bacteria is important in determining its capacity as a biocontrol agent against plant diseases. Screening of candidate strains for antibiotic production, followed by direct detection of their antibiotic profiles, provides a rapid approach in comparison with the traditional method of selection (de Souza and Raaijmakers, 2003). Different analytical techniques for chemical profiling of lipopeptides have been applied: high performance liquid chromatography (HPLC), gas chromatography (GC MS), capillary chromatography hyphenated with mass spectrometry (MS) and UV/Vis spectroscopy (Smyth et al., 2010), nuclear magnetic resonance (NMR) spectroscopy and liquid chromatography-mass spectrometry (LC-MS; Son et al., 2016). Furthermore, matrix-assisted laser desorption ionization—time of flight mass spectrometry (MALDI-TOF MS) has proven to be very effective in the detection and identification of antimicrobial substances, as well as in the analysis of lipopeptide molecules from various extracts, crude culture filtrates, and whole cells (Vater et al., 2002; Athukorala et al., 2009; Cawoy et al., 2015). Despite the speed, low cost and simplicity, there are a limited number of studies related to the separation of lipopeptides by thin-layer chromatography. Based on the *R*_F_-values, high performance thin layer chromatography (HPTLC) can provide information about the polarity, spectral properties (absorbance, fluorescence), and size of the analyte molecules (Morlock and Schwack, 2010). Bioautography is a biological method hyphenated with thin layer chromatography. It is an important tool for the identification of bioactive compounds found in natural products and living organisms. Bioautography is a sensitive, qualitative method and therefore suitable in the initial stages of research, as well as for targeted isolation of compounds (Mãrghitaş et al., 2013).

The aim of this study was to investigate several methods for the fast and reliable detection of *Bacillus* strains that can produce metabolites with potential use in biocontrol. In determining the most efficient method for extracting lipopeptides, MALDI-TOF MS was used, as very fast and effective technique for the identification of distinct lipopeptide compounds within extracts. To identify which lipopeptide compound actually possesses antimicrobial potential, a bioautography assay after separation on the HPTLC chromatograms was used. Antibiotics of biological origin often came as mixtures that appear to exploit synergism as a mechanism of action (Patel et al., 2014). Also, as several studies have suggested (Romero et al., 2007; Ongena and Jacques, 2008), lipopeptides can act in a synergistic manner. Potential synergistic effects of mixtures of individual extracts were also investigated in this study. In our previous research it was shown that lipopeptide extracts of the *Bacillus* strains used in this study exhibit very strong antimicrobial activity (Dimkić et al., 2013; Stević et al., 2014). Therefore, this study is aimed at providing specific information regarding lipopeptide production, as well as solid proof of the strong antibacterial activity of individual lipopeptide compounds in direct antagonism against two pathovars of very important plant pathogens: *Pseudomonas syringae* pv. *aptata* and *Xanthomonas arboricola* pv. *juglandis*.

## Materials and methods

### Bacterial isolates and culture conditions

In this study five strains of the genus *Bacillus* (SS-10.7, SS-12.6, SS-13.1, SS-27.2, and SS-38.4), proven to have antimicrobial activity, were selected for study from the large collection belonging to the Laboratory of Microbiology, Faculty of Biology, University of Belgrade. Four of the strains were originally isolated from soil samples (SS-10.7, SS-12.6, SS-13.1, and SS-38.4), and one from manure (SS-27.2). All are from different locations in Serbia. The method used for isolation of *Bacillus* strains was that described by Berić et al. (2012). In short, after thermal inactivation of vegetative cells (80°C for 10 min), samples were incubated at 30°C for 48 h on Luria–Bertani (LB) agar plates and distinct single colonies were preliminarily characterized by microscopic appearance, Gram staining and catalase testing. The cells were grown in LB broth under aerobic conditions at 30°C for 24 h and used for further experiments.

### Bacterial isolates used in bioautography assay

The antibacterial activity of the *Bacillus* spp. extracts was measured against phytopathogenic bacteria *Pseudomonas syringae* pv. *aptata* (P16) isolated from sugar beets, and *Xanthomonas arboricola* pv. *juglandis* (301, 311, and 320), originating from walnut trees. The phytopathogenic strains were previously identified and belong to the collection of the Laboratory of Microbiology, Faculty of Biology, University of Belgrade. The efficacy of the extracts was measured against *Listeria monocytogenes* (ATCC 19111), the most sensitive strain found in our previous studies. The bacterial strains were cultured overnight at 30°C in LB (HiMedia, India), with the exception of *L. monocytogenes*, which was cultured in Brain-Heart Infusion (BHI) broth (Biomedics, Spain). Suspensions were adjusted to McFarland standard turbidity (0.5), which corresponds to 10^8^ CFU/mL. *X. arboricola* pv. *juglandis* (hereinafter referred to: *X*. *arboricola*) and *P. syringae* pv. *aptata* (hereinafter referred to: *P*. syringae) were grown at 30°C, and *L. monocytogenes* at 37°C.

### Biochemical and phylogenetic analysis

Biochemical identification of *Bacillus* spp. was performed using API 50 CHB and API 20 E commercial kits according to the manufacturer's protocol (BioMérieux, France). The results were read by Apiweb TM software (BioMérieux, France).

Genomic DNA from the *Bacillus* spp. was isolated according to the method described by Le Marrec et al. (2000), and as used in Dimkić et al. (2013). A 16S rRNA gene sequence from each of the isolates was determined from PCR-amplified fragments. The 16S rRNA gene (1,500 bp) was amplified using universal primers UN1_16s_F (GAGAGTTTGATCCTGGC) and UN1_16s_R (AGGAGGTGATCCAGCCG). PCR amplification was performed in a 25 μl reaction mixture containing 0.1–1 μg of template DNA; 25 mM MgCl_2_ at a final concentration of 2.5 mM; a 200 μmol/L concentration of each dNTP; 1 μL of each primer; and 1 U of Taq polymerase (Fermentas UAB, Lithuania). The PCR reactions were performed with an initial denaturation step at 94°C for 5 min, followed by 30 cycles of 94°C for 30 s, 50°C primer annealing for 1 min and a 72°C extension for 30 s, followed by a final extension step at 72°C for 7 min. The PCR products were purified using a column of QIAquick PCR Purification KIT/250 (QIAGEN GmbH, Hilden, Germany) and sent for sequencing to Macrogen sequencing service (Netherlands). The sequences thus obtained were searched for homology with previously sequenced genes in the GenBank database, using the National Center for Biotechnology Information's Blast search program for nucleotides (**BLASTN, RRID: SCR_001598)**. To secure taxonomic relevance, the most related sequences of strain types were used for phylogenetic analyses. All sequences were aligned using ClustalW multiple sequence alignment, and phylogenetic trees were constructed in MEGA 6 (**MEGA Software, RRID: SCR_000667)** using the Neighbor-joining method based on a pair-wise distance matrix with the Kimura two-parameter nucleotide substitution model. The topology of the trees was evaluated by the bootstrap resampling method with 1,000 replicates. The sequences were submitted to the NCBI database and GenBank accession numbers were received.

### Isolation of lipopeptides by two methods of extraction

Each of the *Bacillus* strains was grown in 1000 mL of LB broth with vigorous shaking for 24 h at 30°C. The total volume was used for the purification of lipopeptides from each strain. The cells were removed by centrifugation (5,000 × *g*, 20 min) at 4°C, and cell-free supernatant from the cultures was used for extraction of lipopeptides with ethyl acetate. The culture supernatant and ethyl acetate were mixed in a 1:1.1 volume ratio (*v/v*), with the addition of NaCl (30 g/L). The suspension was mixed for 2 h on a magnetic mixer. The ethyl acetate fraction was collected and dried to completion in a rotary evaporator (Büchi Rotavapor R-215, Switzerland). The metabolites were resuspended in methanol and filtered through a 0.45 μm Durapore™ filter (Milipore, Billerica, USA).

The second approach to extraction involved combining acidic precipitation and solvent extraction, as described by Vater et al. (2002). *Bacillus* strains were grown in the same manner as previously described and equal volumes of prepared supernatant were distributed into several small vessels. Acidification by addition of concentrated HCl to pH 2.0 was performed and precipitation was allowed overnight at 4°C. After centrifugation (5,000 × g, 20 min), the resulting pellet was extracted with methanol under continuous magnetic stirring for a period of 2 h. The obtained extracts were sterilized by filtration through 0.45 μm Durapore™ filters and dried to completion in a rotary evaporator. The metabolites were resuspended in methanol.

The cultivation and extraction for each strain was performed twice, under identical conditions.

### MALDI-TOF MS analysis

Ethyl acetate, methanol, and aqueous extracts obtained from the supernatants of five *Bacillus* spp. isolates were subjected to MALDI-TOF MS analysis. Uninoculated broth extracts were used as negative controls for both extraction methods. The experiments were carried out on a Voyager-DE PRO MALDI-TOF Biospectrometry Workstation (Applied Biosystems, Foster, CA, USA) equipped with a 337 nm pulsed nitrogen laser, as described previously (Athukorala et al., 2009; Abderrahmani et al., 2011). All tested samples were mixed with an equal volume of a saturated solution of 2,5-dihydroxybenzoic acid (DHB; 5 mg in 1 mL of 70% acetonitrile/0.1% trifluoroacetic acid). The suspensions were vortexed gently to provide homogenous mixtures. Aliquots of these mixtures (1–2 μL) were spotted onto the gold-coated plate and air dried. In this study, positive ion detection and reflector mode operation were used. The potential acceleration at 20 kV was held constant during all measurements. The mass spectra were recorded in the range of 800–1,700 *m/z*. The number of laser shots was 400 per spectrum for each tested sample. The masses of the identified molecules were compared with the exact calculated monoisotopic mass according to a MassLynx Molecular Mass Calculator (Waters, USA) and previously published literature.

### High performance thin layer chromatography (HPTLC) with the agar-overlay bioautographic method

In order to screen the lipopeptide extracts, the HPTLC technique was applied (Das et al., 2008). Samples containing 10 μL each of five lipopeptide ethyl acetate extracts (stock concentration of 14.25 mg/mL) and 5 μL of standard compounds of iturin A and surfactin (stock concentration of 10 mg/mL) were applied to 10 × 10 cm and 20 × 10 cm aluminum HPTLC plates (Art. HX384162, Merck, Darmstadt, Germany) as an 8 mm band by using an Automatic TLC sampler 4 (ATS4, CAMAG, Muttenz, Switzerland). Also, 10 μL of mixtures composed of two lipopeptide extracts (5 μL of one + 5 μL of other), in different combinations, was applied on 20 × 10 cm HPTLC plates. Development was performed with a mixture of chloroform: methanol: water (65:25:4, *v/v/v*) in a twin trough chamber (Symmank et al., 2002). After drying, the plates were immersed in 0.2% ninhydrin (2,2-Dihydroxyindane-1,3-dione)/methanol solution (10 mg/mL) for 5 s and heated to 100°C on a TLC Plate Heater III (CAMAG) for enhancement and confirmation of the presence of peptide components. Image capturing was performed at 254 and 366 nm with a CAMAG video documentation system. Images of the plates were processed with the ImageJ processing program (https://imagej.nih.gov/ij/, Rasband W. National Institutes of Health, USA).

The developed HPTLC plates were prepared for the agar overlay variant of bioautography using the modified method of Valgas et al. (2007). On 5 × 5 cm plates, different volumes (1–5 μL) of standards (iturin A and surfactin) of different concentrations (10–50 μg/mL) were spotted and transferred onto LA plates. LB soft agar (5 mL), previously inoculated with 50 μL (1 × 10^8^ CFU/mL) of the bacteria to be tested (*P. syringae* and *X. arboricola* strains), was spread on HPTLC (5 × 5 cm) and LA plates. Autoclave tape was put around the edges of the plate, forming a 0.5 cm deep mold (10 × 10 cm; 20 × 10 cm). All HPTLC plates with samples were sterilized for 15 min under UV light. LB or BHI soft agar (7 and 14 mL), previously inoculated with 70 and 140 μL (1 × 10^8^ CFU/mL) of the tested bacteria (*P. syringae* and *X. arboricola* strains and *L. monocytogenes*), was spread on 10 × 10 cm and 20 × 10 cm plates, respectively. HPTLC plates were first placed in plastic boxes (25 × 15 × 7 cm) with humid atmospheres and left at 4°C for a minimum of 2 h to ensure better diffusion of antimicrobial substances into the agar layer, and then incubated overnight at 30 and 37°C depending on the growth conditions of the strains tested. After incubation, the plates were sprayed with a solution of 0.2% MTT (Thiazolyl Blue Tetrazolium Bromide, Sigma-Aldrich, USA) and 0.1% Triton X-100 (Sigma-Aldrich, USA) for visualization of inhibition zones, and then incubated for 1–2 h.

## Results

### Biochemical and phylogenetic analysis

**Figure 1:**
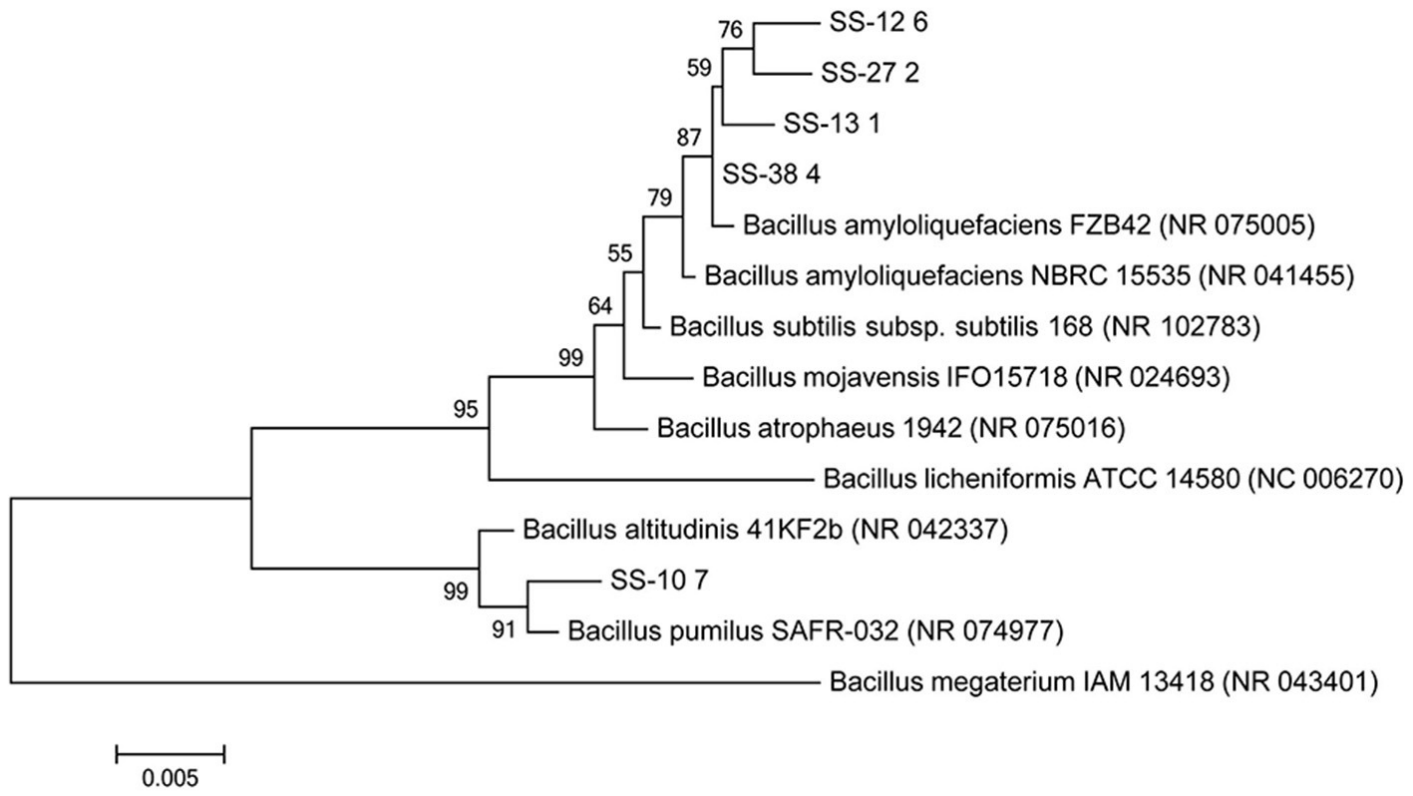
**Neighbor-joining phylogenetic tree based on 16S rDNA sequences (1,500 bp) showing the relationship of the tested isolates (SS-10.7, SS-12.6, SS-13.1, SS-27.2, and SS-38.4) and related reference strains of the genus *Bacillus***. *Bacillus megaterium* (NC_043401) was used as an outgroup. “Bootstrap” values (expressed as a percentage of 1,000 repetitions) >50 are displayed at points of branching. The horizontal bar indicates a genetic distance of 0.005.

Identification of the five *Bacillus* spp. isolates performed by biochemical and enzymatic tests according to API 20 E and 50 CHB identification kits, as well as by BLAST*n* analysis based on a 16S rDNA, is shown in Table S1. Phylogenetic reconstruction based on nucleotide sequences of isolates SS-12.6 (KY780586), SS-13.1 (KY780588), SS-27.2 (KY780587), and SS-38.4 (KY780589) linked them to *Bacillus amyloliquefaciens* FZB42 (NR_075005) and was supported by high bootstrap values, while the SS-10.7 (KY780585) isolate formed a single branch and was most similar to *Bacillus pumilus* SAFR-032 (NR_074977), as shown in Figure 1.

### MALDI-TOF MS analysis

Five lipopeptide-producing *Bacillus* strains (SS-10.7, SS-12.6, SS-13.1, SS-27.2, and SS-38.4) were grown under pre-established conditions in LB medium for 24 h and their extracts obtained either by ethyl acetate extraction or by a combination of acid precipitation followed by methanol extraction. The extracts were then subjected to MALDI-TOF MS analysis. The supernatants were used as aqueous solutions. As a negative control of aqueous solutions, LB medium, whose characteristics were analyzed under the same conditions, was used in order to eliminate medium-derived peaks detected in the spectra of supernatants of the tested isolates (Figure S1). By observing the LB medium spectrum, it can be seen that the detected peaks have masses very similar to lipopeptide compounds. Also, relatively high intensity peaks at *m/z* = 861, 882, 900, 912, 934, 950, 956, 1,016, 1,024, 1,042, 1,048, 1,064, 1,070, 1,102, 1,123, 1,145, 1,174, 1,196, 1,508, and 1,527, which were found in the spectra of certain samples, especially of supernatants of SS-12.6 and SS-13.1, were therefore not taken into further consideration. In contrast, controls, e.g., the uninoculated media in both extraction methods, did not show any contamination by substances from medium components.

**Figure 2:**
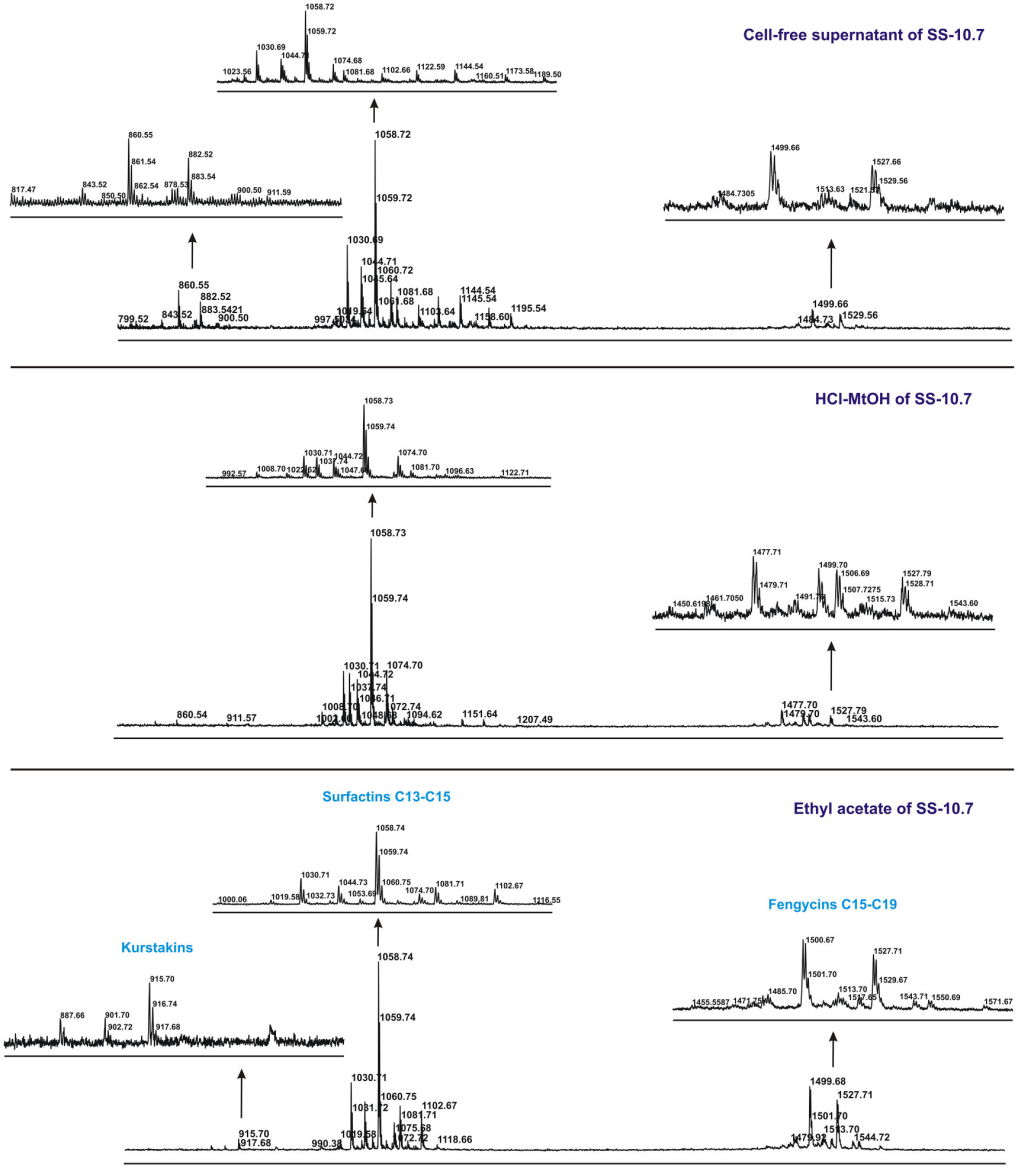
**MALDI-TOF mass spectra of the cell-free supernatant and methanol and ethyl acetate extracts obtained from SS-10.7 in the *m/z* range from 800 to 1,700, in spectrum centroid sections of analyzed peak grouping of presumed lipopeptide compounds**.

The mass spectra of all the extracts studied, particularly of isolates SS-10.7, SS-27.2, and SS-38.4, clearly showed differentiation into three distinct groups of peaks within the mass ranges of *m/z* = 850–950, *m/z* = 1,000–1,150 and *m/z* = 1,450–1,550. The mass spectra signal intensities of the cell-free supernatant and methanol and ethyl acetate extracts of isolate SS-10.7 (Figure 2), with the presumed lipopeptide compounds, are shown in Table 1. The peaks observed in both extracts and supernatant at *m/z* = 1,030, 1,044, and 1,058 differ by 14 Da (-CH_2_-), indicating a series of molecules with different lengths of fatty acid chains. As shown in Table 1, the peaks are attributed to sodium adducts of surfactin C13, C14, and C15, respectively. The peak at *m/z* = 1,058 that corresponds to surfactin C15 was clearly the most abundant in the spectrum. The rest of the observed peaks in this *m/z* area are assigned to the same molecules as protonated or potassium adducts, with the exception of the peaks observed at *m/z* = 1,067, 1,081, and 1,095 which were attributed to potassium adducts of iturin A C13, C14, and C15, respectively. The peaks observed in both extracts and supernatant at *m/z* = 1,499, 1,513, and 1,527 differ by 14 Da (-CH_2_-), and were attributed to sodium adducts of fengycin A C17, C18, and C19, respectively. The peaks at *m/z* = 887, 901, and 915 were detected only in the ethyl acetate extract, and were attributed to sodium adducts of kurstakin C10, C11, and C12, respectively. Similar results for the extracts of isolates SS-27.2 and SS-38.4 were observed (Figures S2, S3). Lipopeptide mixtures of those extracts were mostly composed of surfactin and fengycin adducts (Table 1), with an abundant peak at *m/z* = 1,058 in both extracts and supernatants. The observed peaks were assigned to protonated or sodium adducts of surfactin (C13-C15) and fengycin (C15-C19), particularly in ethyl acetate extracts with the highest S/N ratio. The peaks at *m/z* = 1,067 and 1,081 that were attributed to potassium adducts of iturin A C13 and C14 were characteristic of both extracts and supernatants. Also, besides the presumed kurstakin cyclic compound, the peak at *m/z* = 953 was also present only in ethyl acetate extracts.

**Table 1:** **The comparation of lipopeptides presence in cell-free supernatant, methanol and ethyl acetate extracts of *Bacillus* spp. isolates by MALDI-TOF MS**.

**Lipopeptide molecule**	Monoisotopic mass^*^	**Molecular formula**	**SS-10.7**			**SS-27.2**			**SS-38.4**			**SS-12.6**			**SS-13.1**		
			**SN**	**HCl**	**EA**	**SN**	**HCl**	**EA**	**SN**	**HCl**	**EA**	**SN**	**HCl**	**EA**	**SN**	**HCl**	**EA**
[M+Na]^+^ Kurstakin C10	887.4477	C_38_H_62_N_11_O_12_	**–**	**–**	**×**	ND			ND			ND			ND		
[M+Na]^+^ Kurstakin C11	901.4633	C_39_H_64_N_11_O_12_	**–**	**–**	**×**	ND			**–**	**–**	**×**	ND			ND		
[M+Na]^+^ Kurstakin C12	915.4790	C_40_H_66_N_11_O_12_	**–**	**–**	**×**	**–**	**–**	**×**	**–**	**–**	**×**	ND			ND		
[M+H]^+^ Surfactin C13	1008.6596	C_51_H_89_N_7_O_13_	**–**	**×**	**–**	**–**	**×**	**×**	**–**	**×**	**×**	**–**	**–**	**×**	ND		
[M+H]^+^ Surfactin C14	1022.6753	C_52_H_91_N_7_O_13_	**–**	**×**	**–**	**–**	**×**	**×**	**–**	**×**	**×**	**×**	**–**	**×**	**×**	**–**	**×**
[M+H]^+^ Surfactin C15	1036.6909	C_53_H_93_N_7_O_13_	**–**	**×**	**–**	**–**	**×**	**×**	**–**	**×**	**×**	**×**	**–**	**×**	**–**	**–**	**×**
[M+Na]^+^ Surfactin C13	1030.6416		**×**	**×**	**×**	**×**	**×**	**×**	**×**	**×**	**×**	**–**	**–**	**×**	ND		
[M+Na]^+^ Surfactin C14	1044.6572		**×**	**×**	**×**	**×**	**×**	**×**	**×**	**×**	**×**	**×**	**–**	**×**	ND		
[M+Na]^+^ Surfactin C15	1058.6729		**×**	**×**	**×**	**×**	**×**	**×**	**×**	**×**	**×**	**–**	**×**	**×**	ND		
[M+K]^+^ Surfactin C13	1046.6155		ND			ND			ND			ND			**×**	**–**	–
[M+K]^+^ Surfactin C15	1074.6468		**×**	**–**	**×**	**×**	**×**	**×**	**×**	**×**	**×**	**–**	**–**	**×**	ND		
[M+H]^+^ Iturin A C14	1043.5525	C_48_H_74_N_12_O_14_	ND			ND			ND			ND			**×**	**–**	**×**
[M+Na]^+^ Iturin A C14	1065.5345		**–**	**–**	**×**	ND			**×**	**×**	**×**	ND			ND		
[M+Na]^+^ Iturin A C16	1093.5658	C_50_H_78_N_12_O_14_	**–**	**×**	**–**	ND			ND			ND			ND		
[M+K]^+^ Iturin A C13	1067.4928	C_47_H_72_N_12_O_14_	**×**	**×**	**×**	**×**	**×**	**×**	**×**	**×**	**×**	**–**	**–**	**×**	ND		
[M+K]^+^ Iturin A C14	1081.5084		**×**	**×**	**×**	**×**	**×**	**×**	**×**	**×**	**×**	**×**	**–**	**×**	ND		
[M+K]^+^ Iturin A C15	1095.5241	C_49_H_76_N_12_O_14_	**×**	**×**	**×**	ND			ND			ND			ND		
[M+H]^+^ Fengycin A C15	1449.7881	C_71_H_108_N_12_O_20_	ND			**–**	**×**	**–**	ND			**–**	**–**	**×**	ND		
[M+H]^+^ Fengycin A C16	1463.8037	C_72_H_110_N_12_O_20_	**–**	**×**	**–**	**–**	**×**	**×**	**–**	**×**	**×**	**–**	**×**	**×**	ND		
[M+H]^+^ Fengycin A C17	1477.8194	C_73_H_112_N_12_O_20_	**–**	**×**	**×**	**–**	**×**	**×**	**–**	**×**	**×**	**–**	**×**	**×**	ND		
[M+H]^+^ Fengycin A C18	1491.8350	C_74_H_114_N_12_O_20_	ND			**–**	**×**	**×**	**–**	**×**	**×**	**–**	**–**	**×**	ND		
[M+H]^+^ Fengycin A C19	1505.8507	C_75_H_116_N_12_O_20_	**–**	**×**	**–**	**–**	**×**	**×**	**–**	**×**	**×**	**–**	**–**	**×**	ND		
[M+Na]^+^ Fengycin A C15	1471.7700		**–**	**–**	**×**	**–**	**–**	**×**	**–**	**–**	**×**	ND			ND		
[M+Na]^+^ Fengycin A C16	1485.7857		ND			**×**	**–**	**×**	**×**	**×**	**×**	**–**	**×**	**×**	ND		
[M+Na]^+^ Fengycin A C17	1499.8013		**×**	**×**	**×**	**×**	**–**	**×**	**×**	**×**	**×**	**–**	**–**	**×**	ND		
[M+Na]^+^ Fengycin A C18	1513.8170		**×**	**×**	**×**	**×**	**–**	**×**	**×**	**×**	**×**	**–**	**–**	**×**	ND		
[M+Na]^+^ Fengycin A C19	1527.8326		**×**	**×**	**×**	**×**	**–**	**×**	**×**	**–**	**×**	**–**	**–**	**×**	ND		
[M+K]^+^ Fengycin A C19	1543.8065		**×**	**–**	**×**	**×**	**–**	**–**	**–**	**–**	**×**	ND			ND		

* *Calculated monoisotopic mass according to MassLynx Molecular Mass Calculator (Waters, USA); SN, cell-free supernatant; HCL, HCl-methanol extract; EA, ethyl acetate extract; (–), a molecule was not present in particular extract; (**×**), a molecule was present in particular extract; (ND), a molecule was not detected for particular strain*.

In the analysis of the spectra of supernatants and both types of extracts of isolates SS-12.6 and SS-13.1, somewhat different results were obtained (Table 1). Specifically, when analyzing the spectra of the supernatants of these isolates it became clear that the most abundant peaks match the ones observed in the negative control (Figure S1). In addition, only the peak at *m/z* = 1,023 was recorded in both supernatants. Obvious differences between ethyl acetate and methanol extracts, in terms of the variety of proposed lipopeptide compounds, were also observed. Only for the ethyl acetate extracts can a characteristic distribution of the peaks be seen according to the principle of the aforementioned *m/z* grouping of lipopeptides. For both isolates the occurrence of kurstakins, as well as fengycins for the isolate of SS-13.1, was not detected. The peaks observed for the SS-12.6 isolate were attributed to protonated or sodium adducts of surfactin (C13-C15), with the most prominent peak at *m/z* = 1,058, and fengycin molecules (C16-C19), though only in ethyl acetate extracts with the highest S/N ratio (Figure S4). The peaks at *m/z* = 1,067 and 1,081, which were attributed topotassium adducts of iturin A C13 and C14, were characteristic of ethyl acetate extracts. The mass spectrum of ethyl acetate extract from SS-13.1 showed only protonated adducts of surfactin (C14 and C15) and iturin A C14. Peaks at *m/z* = 1,000, 1,020, and 1,054 were also observed.

### HPTLC profiles and antimicrobial activity of *Bacillus* spp. extracts

**Figure 3:**
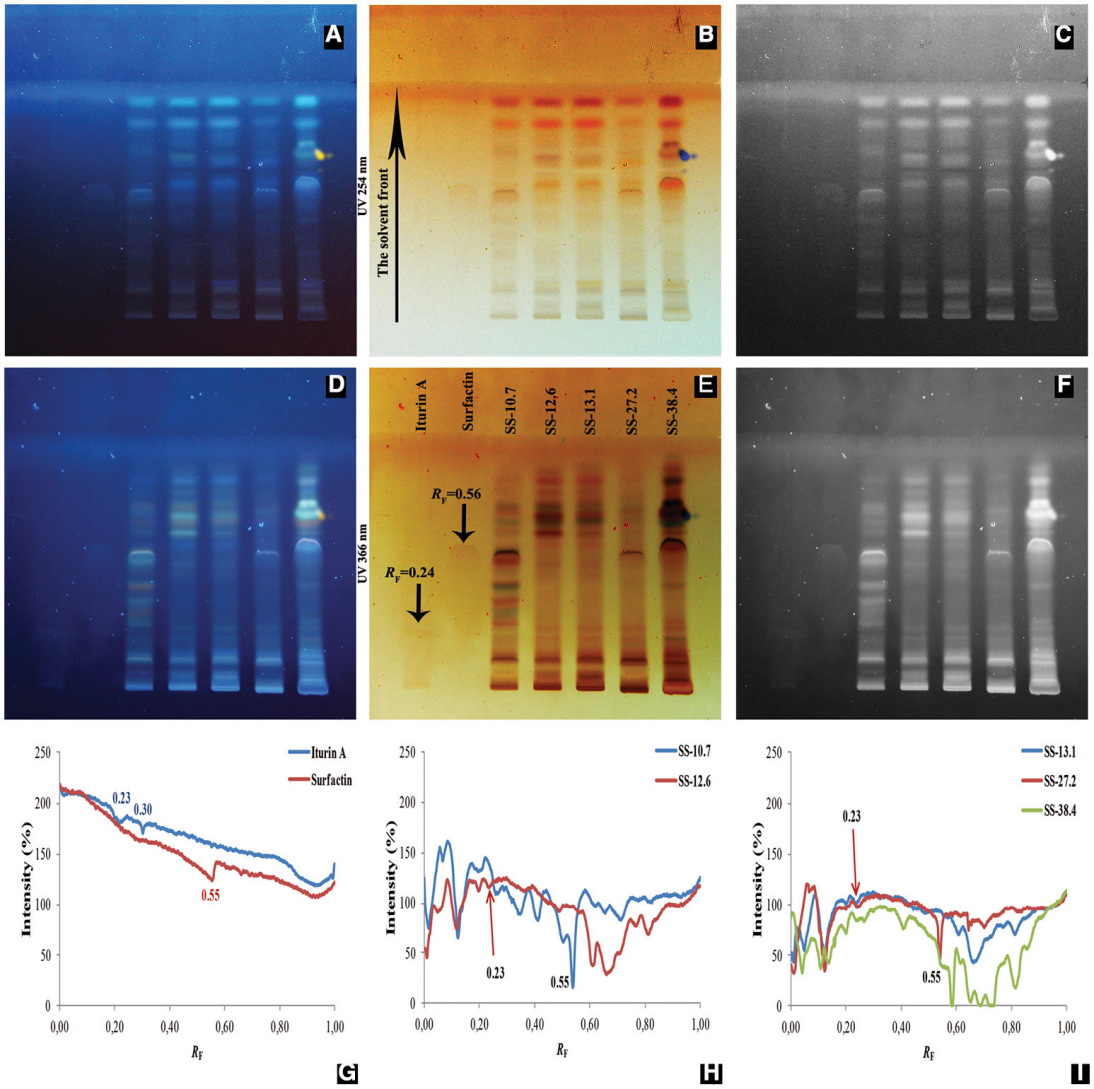
**Analyzed chromatograms of the standard compounds (iturin A and surfactin) and five *Bacillus* ethyl acetate extracts (SS-10.7, SS-12.6, SS-13.1, SS-27.2, and SS-38.4) under UV light at 254 nm (A–C)** and 366 nm **(D–F)** in the form of regular **(A,D)**, inverted **(B,E)** and photographs in grayscale mode **(C,F)**. Graphical display of intensity zones (expressed in pixels) as a function of distance passed of iturin A and surfactin **(G)**; extracts of isolates SS-10.7 and SS-12.6 **(H)**; and extracts of isolates SS-13.1, SS-27.2, and SS-38.4 **(I)**, are shown.

In this study, HPTLC was applied in order to compare complex mixtures of lipopeptide extracts obtained from five *Bacillus* isolates. Furthermore, the antimicrobial activity of lipopeptides separated on the HPTLC chromatograms was recorded by the agar overlay method. Iturin A and surfactin were used as lipopeptide standards. Visualization of the HPTLC chromatograms was performed at wavelengths of 254 and 366 nm. The line profiles of HPTLC chromatograms for ethyl acetate extracts and standards are presented graphically (Figure 3).

As shown in Figure 3, there is a difference in signal intensities of standard compounds at both detection wavelengths, as well as one observable signal at 366 nm for iturin A. In Figure 3G, two maximum intensities for iturin A with *R*_F_-values of 0.23 and 0.30 are shown, whereas surfactin displayed a single peak, with an *R*_F_-value of 0.55. After analysis was conducted of the extracts, peaks were observed with *R*_F_-values of 0.23 for SS-12.6 (Figure 3H), SS-13.1 and SS-27.2, and one with a slightly higher value (*R*_F_ = 0.27) for the SS-38.4 extract (Figure 3I). The most prominent peaks, with *R*_F_-values equivalent to the surfactin standard, were detected in extracts of isolate SS-10.7 (Figure 3H) and SS-27.2 (Figure 3I). In the SS-38.4 extract, a peak with *R*_F_-value 0.58 was also detected, similar to the value of the distance passed by the surfactin standard. Furthermore, as shown in Figure 3I, samples such as SS-12.6, SS-38.4, and SS-13.1 have characteristic peaks with *R*_F_-values between 0.60 and 0.80. Sample SS-10.7 contained a specific peak at *R*_F_-value 0.10. In order to identify the chromatographic zones which correspond to peptide compounds, the chromatograms were derivatized by immersion in a ninhydrin solution. All extracts were positive for ninhydrin reaction, which clearly indicates the presence of (lipo)peptide compounds in the extracts of the tested isolates, and the iturin A standard showed several stripes with different *R*_F_-values (Figure S5).

**Figure 4:**
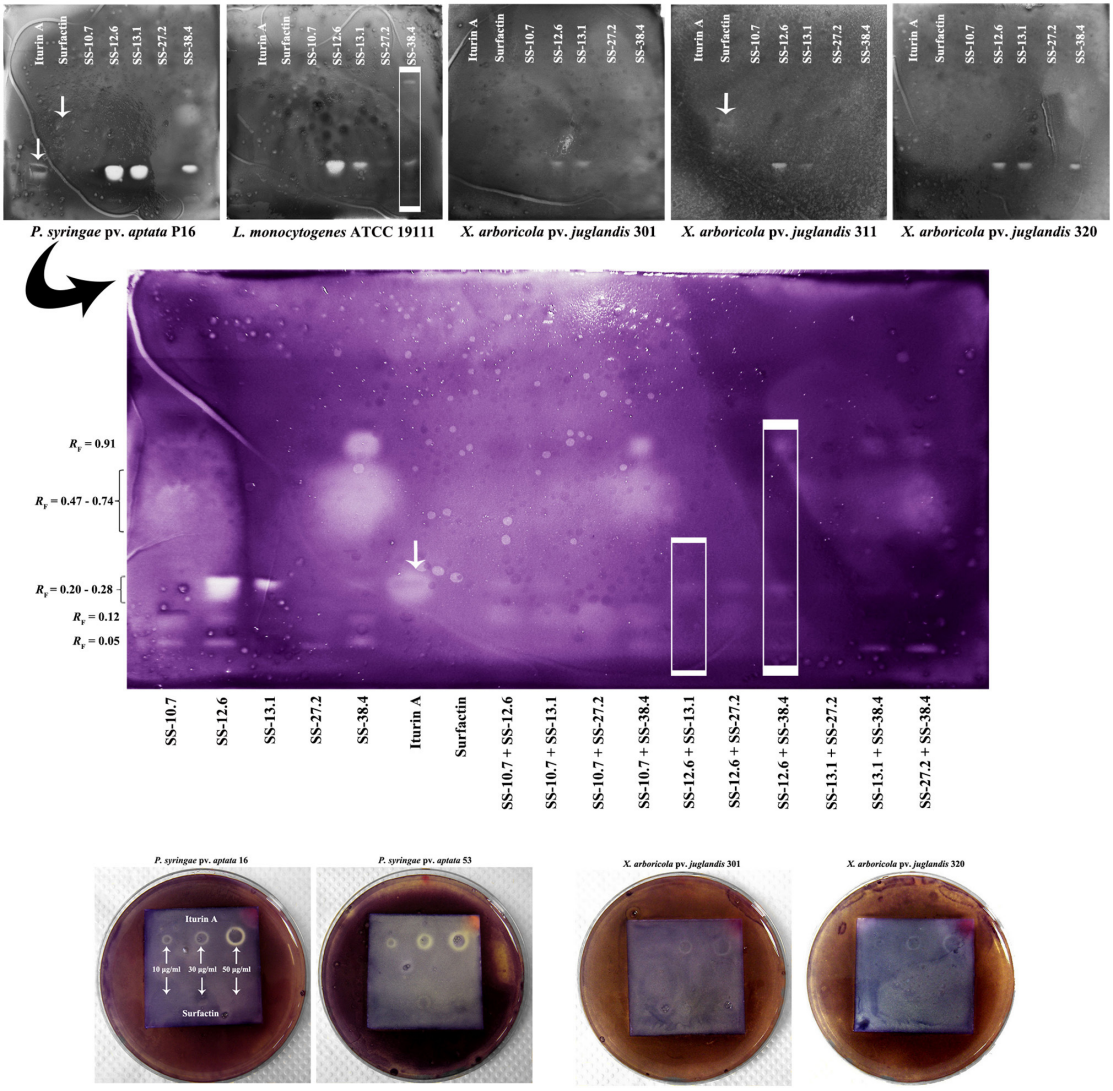
**Bioautography analysis of the standard compounds and *Bacillus* ethyl acetate extracts, individually and in combinations, against selected indicator strains**. The arrows on the chromatograms indicate obtained or expected retention factors (*R*_F_) of standard compounds; and rectangles observed differences. Obtained *R*_F_-values are indicated on the left side of the central chromatogram.

The results of bioautography tests of antimicrobial activity for the individual extracts against indicator strains (*X*. *arboricola, P*. *syringae*, and *L*. *monocytogenes*) and their mixtures against *P. syringae* pv. *aptata* 16 are shown in Figure 4. It was shown that at concentrations of 10 μg/mL for the iturin A standard, significant inhibition of the *Pseudomonas* pathogens isolated from sugar beet occurred. Growth inhibition was not observed for the surfactin standard in the range of tested concentrations, except in the case of *X. arboricola* 311. The extracts of SS-12.6, SS-13.1, and SS-38.4 showed the largest inhibition zones, generally. The extract of SS-27.2 inhibited the growth of *L. monocytogenes*. Extracts of SS-10.7 and SS-27.2 exhibited the weakest effect against all tested pathogens, with the exception of *P. syringae*. Only one visually striking inhibition zone at *R*_F_-value of 0.05 was noted for extracts of these two strains, and another one at 0.12 for SS-10.7. The zone of inhibition for *P. syringae* 16 at *R*_F_-values between 0.47 and 0.74 was observed only for the SS-38.4 extract (Figure 4).

The results of bioautography tests of antimicrobial activity for the individual extracts in different mixtures showed that there was no synergistic effect or stronger antimicrobial activity of mixtures. The combinations of the strongest individual antagonists (SS-12.6 and SS-13.1, or SS-12.6 and SS-38.4), shown in Figure 4 (in the framed rectangles), demonstrated weak or no antimicrobial activity, in comparison with zones obtained by individual extracts. The inhibition zones were minimized or totally eliminated in the case of these combinations.

## Discussion

Two different methods of lipopeptide extraction from supernatants of producing strains were used. By using mass spectrometry an attempt was made to determine which method of extraction yielded more of the expected lipopeptides. In this way, information about secondary metabolites produced by a microorganism can be obtained very quickly and with high precision, without the need for purification of the detected compounds. The classification of lipopeptides into different classes was made according to calculated monoisotopic masses and previously published literature. The cultivation and extractions for each strain were performed twice, but given that the resulting spectra were almost identical, one was chosen for further analysis. Analysis of the mass spectra of all the extracts tested, particularly extracts of isolates SS-10.7, SS-27.2, and SS-38.4, clearly showed distinct groups of peaks at *m/z* = 850–950 which correspond to the kurstakin family, *m/z* = 1000–1150 which includes representatives of the surfactin and iturin families, and *m/z* = 1,450–1,550 which represents the fengycin family. Based on the spectra, the overall yield, i.e., relative intensity of the peaks, was higher for extracts obtained by ethyl acetate extraction. Methanol extraction was less efficient. The relative intensities of peaks in the spectra of culture supernatants were very low, given that lipopeptides were not extracted. However, testing of supernatants may indicate the presence of certain lipopeptide compounds and serve as a quick screening of isolates for further extraction. Although MALDI-TOF MS is only a semi-quantitative analytical technique, the values obtained for relative signal intensities provide useful information regarding the abundance of the analyzed molecules in each extract, without the need for absolute quantitation. Surfactins were detected in higher amounts in all extracts, compared to iturin and fengycin, and similar observations were reported recently by Cawoy et al. (2015). Likewise, the homologs of surfactin are not distributed in the same way through the two types of extracts and the supernatants. The peak at *m/z* = 1,058 that corresponds to surfactin C15 was clearly the highest in the spectrum, suggesting this compound was the most abundant relative to other compounds present in the extracts of isolates SS-10.7, SS-12.6, SS-27.2, and SS-38.4. This abundant molecule was detected in both types of extracts and the supernatants. The same *m/z* value for sodium adduct of surfactin A has been reported previously (Mukherjee and Das, 2005; Velho et al., 2011). Also, the presence of iturin A C14 molecules in almost all extracts and supernatants was observed. The same mass for potassium adduct of iturin A was reported recently (Cawoy et al., 2015). It is interesting to point out that the peaks at the *m/z* value corresponding to cyclic kurstakins were detected only in the ethyl acetate extracts. Also, the peak at *m/z* = 953 was characteristic of the isolates of SS-27.2 and SS-38.4, which can be attributed toa linear form of kurstakin iC14 (Abderrahmani et al., 2011; Béchet et al., 2012). The most prominent peak in the fengycin *m/z* group, according to the ethyl acetate extraction method, can be attributed to a sodium adduct of fengycin C17, with the alanine residue at position 6 of the peptide ring, as reported previously (Vater et al., 2002; Arguelles-Arias et al., 2009). For the extracts of SS-12.6 and SS-13.1 different results were obtained. Namely, only the peak at *m/z* = 1,023, which can be attributed to protonated adduct of surfactin C14 (Mukherjee and Das, 2005; Athukorala et al., 2009), was detected in the supernatants and ethyl acetate extracts of both strains. The homologs of surfactin and fengycin were not distributed the same way among the two types of extracts and supernatant of SS-12.6. However, the ethyl acetate extract of SS-12.6 gave nearly identical results to those previously obtained by Orbitrap mass spectrometry analysis (Dimkić et al., 2013). Surely it can be considered that this strain produces surfactin and iturin, as well as forms of fengycin compounds, which only became evident after testing the ethyl acetate extract. Peaks of the highest relative intensity for SS-13.1 remained uncharacterized, since for the given values (*m/z* = 1,000, 1,020, and 1,054) there was no overlap with the literature data. The closest value (*m/z* = 1053.6) has been reported for the sodium adduct of bacillomycin D C14 (Athukorala et al., 2009). For both extracts and the supernatant of SS-13.1, no peak grouping was detected in the mass range of the fengycin family, which confirmed our previous results from PCR screening, where the presence of the *fenD* gene was not shown (Dimkić et al., 2013). When comparing these results with our previous PCR screening (Stanković et al., 2012), it becomes clear that most of the isolates actually produce a number of different compounds. Similar observations were reported previously with detection of the biosynthetic operons, but without the occurrence of the appropriate antibiotics in the extracts (Athukorala et al., 2009). These findings confirm the fact that despite the existence of many antibiotic-producing genes in individual isolates, only a few antibiotics can be synthesized in higher concentrations (Mootz et al., 2001). Alternatively, the production of some antibiotics might be delayed compared to others, which can be inferred from the differences in peak intensities (Hofemeister et al., 2004). What has undoubtedly been confirmed for all the isolates tested are groups of peaks indicating iturin and surfactin families with similar intensities, pointing out their dominant presence, as in previous studies (Vater et al., 2002; Ramarathnam et al., 2007; Athukorala et al., 2009). The assumed values of surfactin compounds were found for all tested isolates. The data obtained in this study certainly favor ethyl acetate extraction as the method of choice for further isolation and concentration of lipopeptide compounds. Downstream processing is an important step in major biomolecule production processes and organic extraction from the culture supernatant is a frequent practice for obtaining hydrophobic products (Coronel-León et al., 2015). It has been considered an obstacle to reasonably economical production, since it accounts for 50–80% of total production costs (Mnif et al., 2013). The results are consistent with earlier studies on *Bacillus* isolates which showed that the use of acid precipitation followed by methanol extraction led to a reduction of biosurfactant activities (75%) present in cell-free supernatant, to only 23% of the initial activity (Yakimov et al., 1995). In this respect, ethyl acetate, in which the hydrophobic residues of lipopeptide compounds probably dissolve better than in methanol, is recommended as a relatively low cost solvent that would make a suitable candidate for further research and commercial production.

Another approach of this study has included the use of HPTLC, which combines biological and chemical detection of marker compounds. These tests are often used to demonstrate the activity of the extract or the individual pure substances obtained from living organisms. Since ethyl acetate extracts had higher yield and more diversity of the lipopeptides (kurstakins were extracted only in this way), HPTLC analysis was conducted only with ethyl acetate extracts. Thus, HPTLC bioautography was used as demonstration of the antimicrobial activity of the individual compounds. The extractions for each strain were performed twice, but given that the resulting HPTLC plates were identical, one was chosen for further analysis. The wide diversity of the samples, in terms of the production of different compounds, was confirmed for all of the strains tested. Also, there were evident differences between the extracts of different strains in their signal intensities and *R*_F_-values for certain substances. All HPTLC chromatograms, under different UV light, showed evident differences between the extracts and the standards, for instance the only observable signal at 366 nm was for iturin A. Treatment with ninhydrin should result in the appearance of an intense purple color if the extract contains a peptide component, and it is often used in HPTLC analysis of lipopeptides (Kumar et al., 2009; Gordillo and Maldonado, 2012). It was also interesting that for iturin A, several bands were obtained, for which the manufacturer (Sigma-Aldrich, USA) guaranteed a high rate of purification of compounds (over 95%), but it was probably the mixture of the structurally diverse homologs (Figure S5). Analysis of the extracts revealed values similar to the tested standards. These findings correlate with our previous results (Stanković et al., 2012; Dimkić et al., 2013) which pointed out the presence of iturin operon genes, which, together with MALDI-TOF MS analysis, indicates the presence of a presumed compound that corresponds to iturin A C14. Although the presence of surfactin molecules in all tested strains was confirmed, the results obtained by HPTLC chromatography reveal the existence of this particular homolog only in extracts SS-10.7 and SS-27.2 (Figures 3H,I). Different *R*_F_-values in the extracts of SS-38.4 (0.58), SS-12.6, and SS-13.1 (0.65) indicate structural diversity of these molecules.

According to the results obtained from the bioautography test of standard compounds, it was clear that iturin homologs were responsible for the inhibition the indicator strains of *Xanthomonas* and *Pseudomonas*. Indeed, when considering the effects of the individual extracts it was observable that SS-12.6, SS-13.1, and SS-38.4 extracts had the highest concentrations of iturin, so they achieved the largest inhibition zones. Also, it was shown that the SS-12.6 extract was the most potent, within *R*_F_-values 0.20 and 0.28, indicating a prospective synergism of several homologs of iturin or a greater quantity of one of them. However, as previously observed with ninhydrin reactions, the existence of iturin in multiple bands indicates a high probability that only the appropriate homologs expressed an antibacterial effect. The different activity of particular homologs was observed when applying iturin standards in the form of spots on HPTLC plates, with the central part of each spot having no antibacterial activity, and detected activity against *P. syringae* pv. *aptata* 16 in several cases (Figure 4). Although the presence of surfactin compounds in the extracts was clearly demonstrated in previous analyses, growth inhibition was scarce. The biological role of surfactin is to support the colonization of surfaces and supply nutrients through surface wetting, as well as having detergent properties. But, surfactins also act as antimicrobials; at high concentrations they act as classical detergents and in lower concentrations they can facilitate the formation of ion-dependent pores in the cell membrane (Heerklotz and Seelig, 2007). Substantial literature data point to biocontrol activity of lipopetide compounds from various extracts. Similar *R*_F_-values as in our research for iturin (0.36) and surfactin (0.80) have been reported in studies of lipopeptide extracts obtained from *B. subtilis* UMAF6639 isolate against phytopathogenic *Xanthomonas campestris* pv. *cucurbitae* and *Pectobacterium carotovorum* subsp. *carotovorum* (Zeriouh et al., 2011). Furthermore, the results against phytopathogenic fungi for extracts of *B. amyloliquefaciens* PPCB004, with *R*_F_-values of 0.30 for iturin and between 0.75 and 0.80 for surfactin, were in accordance with our results (Romero et al., 2007; Arrebola et al., 2010a,b). *R*_F_-values of fengycin homologs from 0.08 to 0.20 were also observed in these studies, which coincide with the range of the detected zones in our study for all extracts except that of SS-13.1. Somewhat different *R*_F_-values in various studies might be due to a different composition of the mobile phase, temperature, humidity or instrumental instability (Wong et al., 2014). Considering the investigation of synergistic effects, the advantage of using a different combination of extracts is that a lower concentration of individual agents is required. This approach could have significant economic and environmental impacts if applied on a large scale. A synergistic effect of mixtures was not observed, and the weak inhibition zones detected can be attributed to a possible additive effect of the individual compounds. In addition, combinations of the strongest extracts (SS-12.6 and SS-13.1 or SS-12.6 and SS-38.4), shown in Figure 4 (in the framed rectangles), exhibited minimal inhibition zones or no zones at all, which may indicate an antagonistic effect between active substances in those mixtures. The probable cause of antagonism could be that they have the same target, or even chemical interactions (direct or indirect) between the compounds, where one reduces the activity of another (Bassolé and Juliani, 2012). The genus *Bacillus* contains a very heterogeneous group of species with a cosmopolitan distribution. All of the strains from this research showed potent antimicrobial effects against plant pathogens and, together with other members of the *B. subtilis* group, are considered harmless to humans and safe for use.

Our analysis of the mass spectra of all extracts tested clearly shows distinct peak groupings which correspond to the kurstakin, iturin, surfactin, and fengycin families of compounds. The ethyl acetate extraction method had better yields and a higherdiversity of lipopeptide compounds, including kurstakins. HPTLC bioautography makes it possible to identify the active component from a mixture of lipopeptide extracts in a very effective manner, *in situ*. Antimicrobial effects were achieved by most of the lipopeptide compounds at the same time. However, by far the most effective were iturins. This study presents a good starting point for further research into the fast detection of potential biocontrol strains and their metabolites, particularly during the screening of a large number of natural isolates, taking into account the high prevalence and availability of these beneficial microorganisms worldwide.

## Author contributions

Conceived and designed experiments: ID, SS, MN, MP, PR, DF, and TB. Completed experiments and collected data: ID, MN, and PR. Analyzed and interpreted data: ID, SS, MN, MP, PR, DF, and TB. Wrote, critically revised and approved the final version of the manuscript: ID, SS, MN, MP, PR, DF, and TB.

## Funding

This work was supported by the Ministry of Education, Science and Technological Development of Serbia, Grant No. 173026.

### Conflict of interest statement

The authors declare that the research was conducted in the absence of any commercial or financial relationships that could be construed as a potential conflict of interest.
