# Corrigendum: The Profile and Antimicrobial Activity of *Bacillus* Lipopeptide Extracts of Five Potential Biocontrol Strains

**DOI:** 10.3389/fmicb.2017.01500

**Published:** 2017-08-04

**Authors:** Ivica Dimkić, Slaviša Stanković, Marija Nišavić, Marijana Petković, Petar Ristivojević, Djordje Fira, Tanja Berić

**Affiliations:** ^1^Department of Microbiology, Faculty of Biology, University of Belgrade Belgrade, Serbia; ^2^Department of Physical Chemistry, Institute of Nuclear Sciences “Vinca,” University of Belgrade Belgrade, Serbia; ^3^Innovation Centre of the Faculty of Chemistry Ltd., University of Belgrade Belgrade, Serbia; ^4^Department of Biochemistry and Molecular Biology, Faculty of Biology, University of Belgrade Belgrade, Serbia

**Keywords:** *Bacillus*, lipopeptides, MALDI-TOF MS, HPTLC, iturin, ethyl acetate extract, bioautography

In the original article, there was an error. Due to the oversight, a technical error was made in some parts of first two sentences, in the section *Bacterial Isolates Used in Bioautography Assay*. The corrections include the isolates code (IZB for the *Xanthomonas* strains), and the origin of the collection (Institute for Plant Protection and Environment, Belgrade, Serbia instead Laboratory of Microbiology, Faculty of Biology, University of Belgrade).

The corrected paragraph appears below:

The antibacterial activity of the *Bacillus* spp. extracts was measured against phytopathogenic bacteria *Pseudomonas syringae* pv. *aptata* (P16) isolated from sugar beets, and *Xanthomonas arboricola* pv. *juglandis* (IZB 301, IZB 311, and IZB 320; hereinafter referred to: 301, 311, and 320), originating from walnut trees. The phytopathogenic strains were previously identified and belong to the collection of the Institute for Plant Protection and Environment, Belgrade, Serbia.

The authors apologize for this error and state that this does not change the scientific conclusions of the article in any way.

## Conflict of interest statement

The authors declare that the research was conducted in the absence of any commercial or financial relationships that could be construed as a potential conflict of interest.

