# Supplementary material for: The Profile and Antimicrobial Activity of Bacillus Lipopeptide Extracts of Five Potential Biocontrol Strains
Source: Front Microbiol. 2017 May 23;8:925. doi: 10.3389/fmicb.2017.00925 (PMC5440568; doi:10.3389/fmicb.2017.00925)
Supplement: Supplementary file 1 [file Table1.DOC]

Supplementary Material

**The profile and antimicrobial activity of *Bacillus* lipopeptide extracts of five potential biocontrol strains**

**Dimkić Ivica^1^, Stanković Slaviša^1^, Nišavić Marija^2^, Petković Marijana^2^, Ristivojević Petar^3^, Fira Djordje^4^, Berić Tanja^1*^**

^1^Department of Microbiology, Faculty of Biology, University of Belgrade, Belgrade, Serbia

^2^Institute of Nuclear Sciences”Vinča”, Department of Physical Chemistry, University of Belgrade, Belgrade, Serbia

^3^Innovation Centre of the Faculty of Chemistry Ltd., University of Belgrade, Belgrade, Serbia

^4^Department of Biochemistry and Molecular Biology, Faculty of Biology, University of Belgrade, Belgrade, Serbia

*** Correspondence:**

Tanja Berić, PhD

Faculty of Biology, University of Belgrade

Studentski trg 16, 11000 Belgrade, Serbia

Telephone: +381 11 2637 364

Fax: +381 11 2637 364;

E-mail: tanjab@bio.bg.ac.rs

# Supplementary Data

**Table S1.** Preliminary identification of the five *Bacillus* isolates on the basis of biochemical and enzymatic tests, as well as on BLAST*n* analysis based on 16S rDNA.

**Figure S1.** MALDI-TOF mass spectra of LB medium as negative control in the *m/z* range from 700-1700.

**Figure S2.** MALDI-TOF mass spectra of the cell-free supernatant, methanol and ethyl acetate extracts obtained from SS-27.2. Lipopeptide compounds were detected in the *m/z* range from 800-1700.

**Figure S3.** MALDI-TOF mass spectra of the cell-free supernatant, methanol and ethyl acetate extracts obtained from SS-38.4. Lipopeptide compounds were detected in the *m/z* range from 800-1700.

**Figure S4.** MALDI-TOF mass spectra of the cell-free supernatant, methanol and ethyl acetate extracts obtained from SS-12.6. Lipopeptide compounds were detected in the *m/z* range from 800-1700.

**Figure S5.** The iturin A standard (Sigma-Aldrich, USA) with purification rate over 95% and several stripes with different *R*_F_ values.

**Table S1.**

| Isolate | The percentages based on the identification of the biochemical analysis (API 20 E and 50 CHB) | |  | The closest reference strain from the NCBI base and achieved maximum of identity (%) by 16S rDNA sequences | |
| --- | --- | --- | --- | --- | --- |
|  |  |  |  |  |  |
| SS-10.7 | *Bacillus subtilis/amyloliquefaciens* | 90.0 |  | *Bacillus pumilus* SAFR-032 (NR_074977) | 98.86 |
|  |  |  |  | *Bacillus safensis* FO-036b (NR_041794) | 98.77 |
|  |  |  |  | *Bacillus stratosphericus* 41KF2a (NR_042336) | 98.51 |
|  |  |  |  |  |  |
| SS-12.6 | *Bacillus subtilis/amyloliquefaciens* | 94.5 |  | *Bacillus amyloliquefaciens* FZB42 (NR_075005) | 99.13 |
|  | *Bacillus licheniformis* | 5.8 |  | *Bacillus subtilis* subsp. *subtilis* 168 (NR_102783) | 98.90 |
|  |  |  |  | *Bacillus vallismortis* DSM11031 (NR_024696) | 98.82 |
|  |  |  |  |  |  |
| SS-13.1 | *Bacillus subtilis/amyloliquefaciens* | 98.9 |  | *Bacillus amyloliquefaciens* FZB42 (NR_075005) | 99.63 |
|  |  |  |  | *Bacillus subtilis* subsp. *subtilis* 168 (NR_102783) | 99.62 |
|  |  |  |  | *Bacillus vallismortis* DSM11031 (NR_024696) | 99.24 |
|  |  |  |  |  |  |
| SS-27.2 | *Bacillus subtilis/amyloliquefaciens* | 94.7 |  | *Bacillus amyloliquefaciens* FZB42 (NR_075005) | 99.04 |
|  |  |  |  | *Bacillus subtilis* subsp. *subtilis* 168 (NR_102783) | 98.77 |
|  |  |  |  | *Bacillus vallismortis* DSM11031 (NR_024696) | 98.69 |
|  |  |  |  |  |  |
| SS-38.4 | *Bacillus amyloliquefaciens* | 81.9 |  | *Bacillus amyloliquefaciens* FZB42 (NR_075005) | 99.60 |
|  | *Bacillus licheniformis* | 13.5 |  | *Bacillus subtilis* subsp. *subtilis* 168 (NR_102783) | 99.36 |
|  | *Bacillus subtilis* | 4.3 |  | *Bacillus vallismortis* DSM11031 (NR_024696) | 99.28 |
|  |  |  |  |  |  |
|  |  |  |  |  |  |
