# Supplementary figures and images for: The Profile and Antimicrobial Activity of Bacillus Lipopeptide Extracts of Five Potential Biocontrol Strains
Source: Front Microbiol. 2017 May 23;8:925. doi: 10.3389/fmicb.2017.00925 (PMC5440568; doi:10.3389/fmicb.2017.00925)

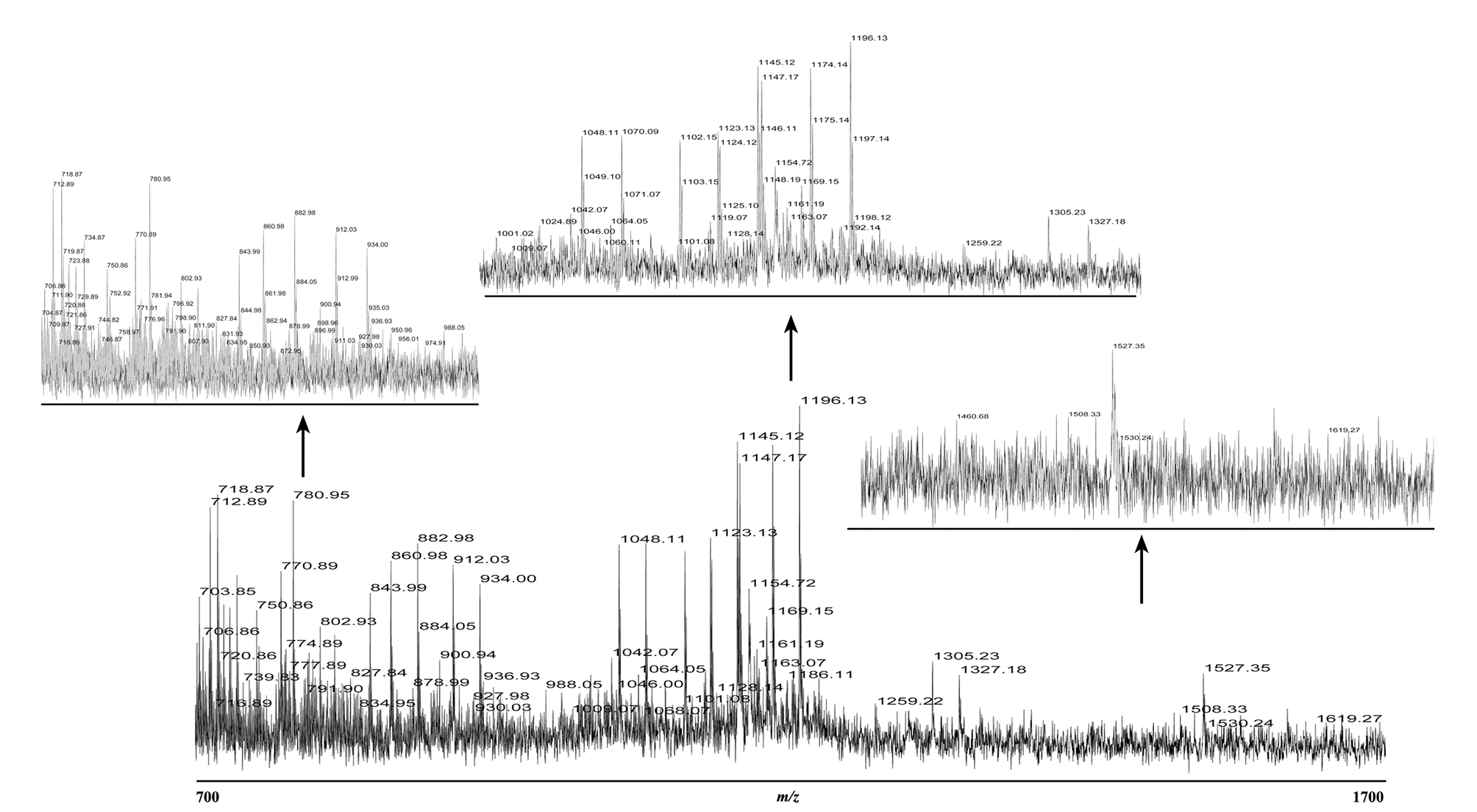

Supplement: Supplementary file 2 [file Image1.TIF]

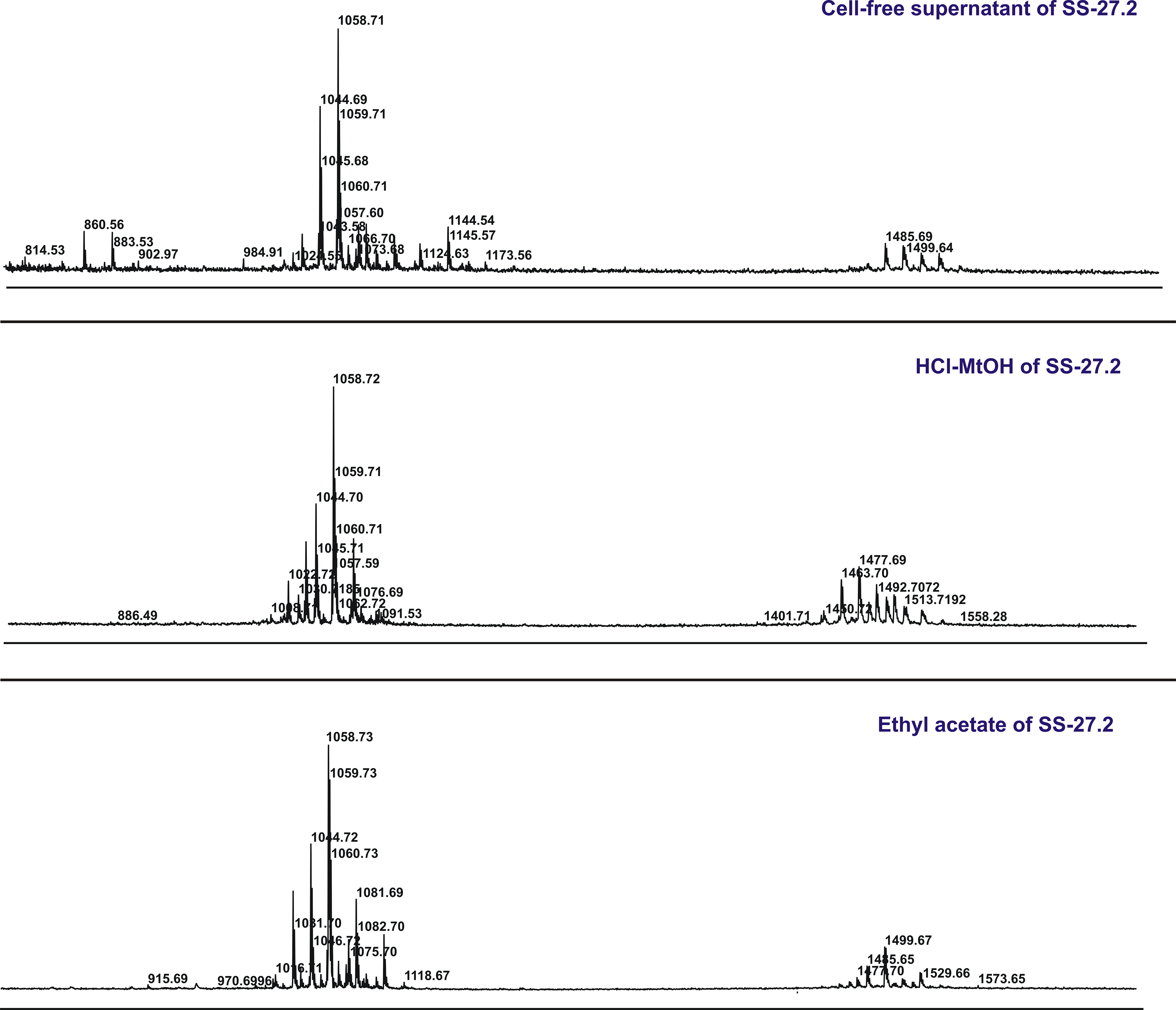

Supplement: Supplementary file 3 [file Image2.TIF]

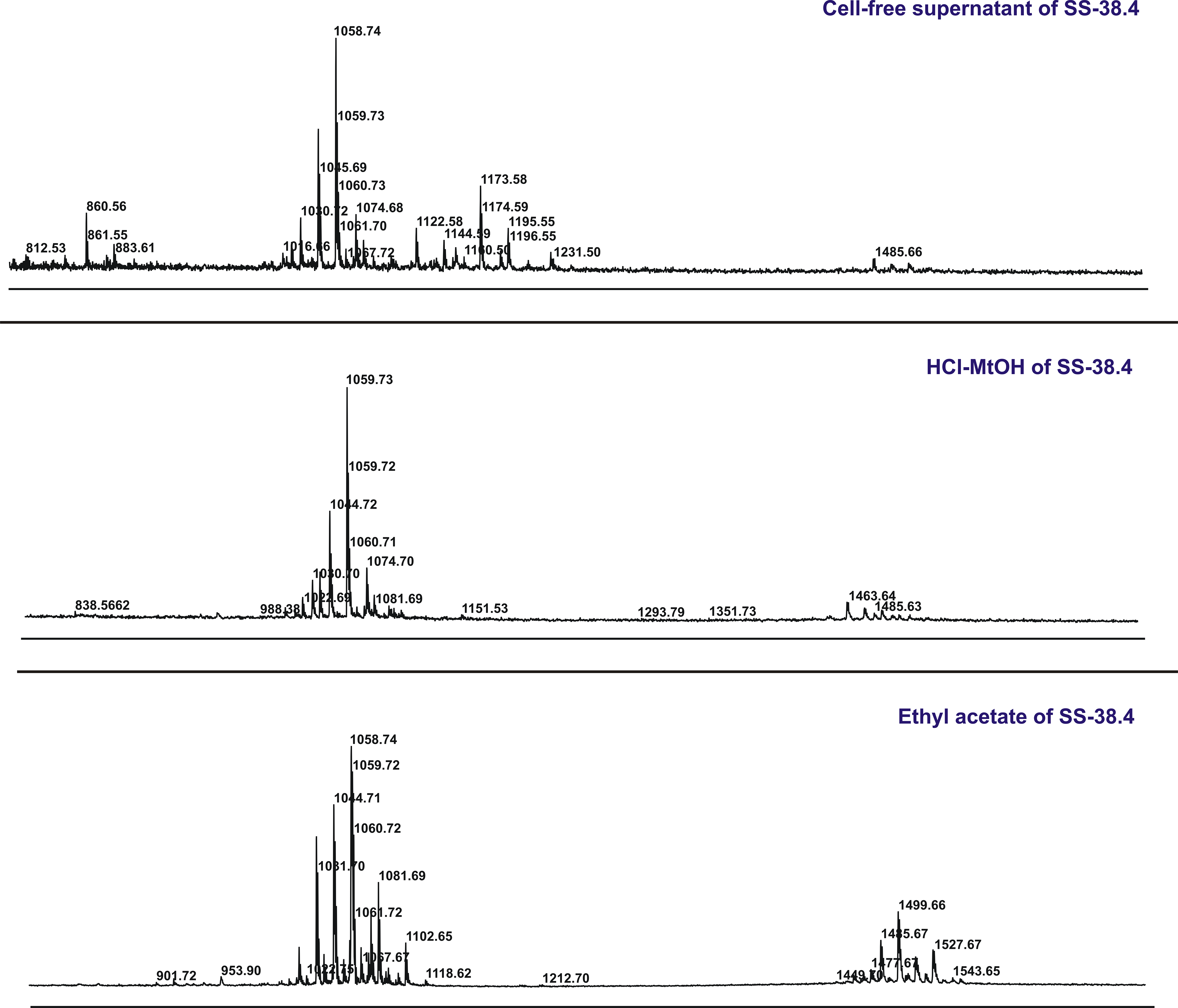

Supplement: Supplementary file 4 [file Image3.TIF]

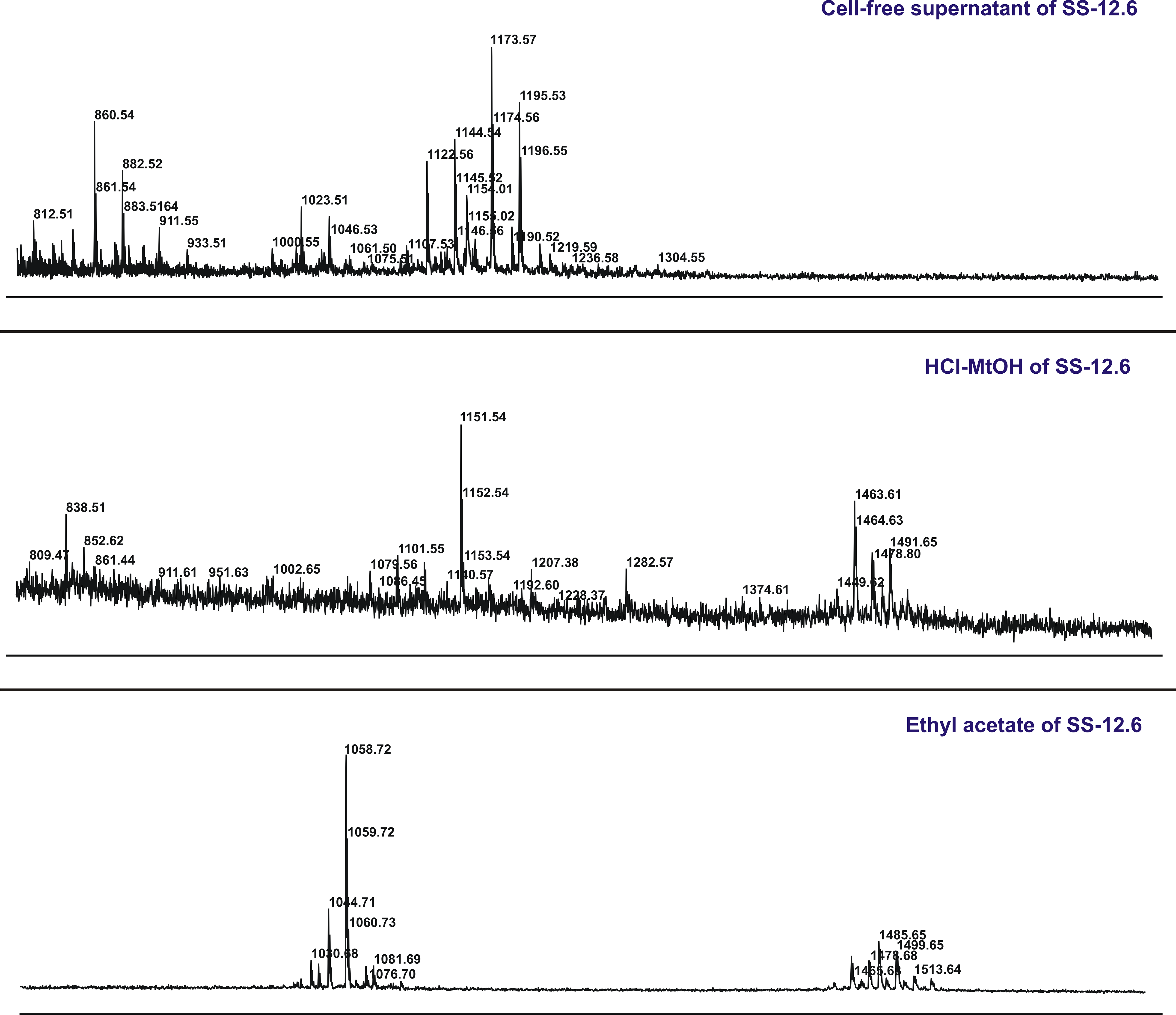

Supplement: Supplementary file 5 [file Image4.TIF]

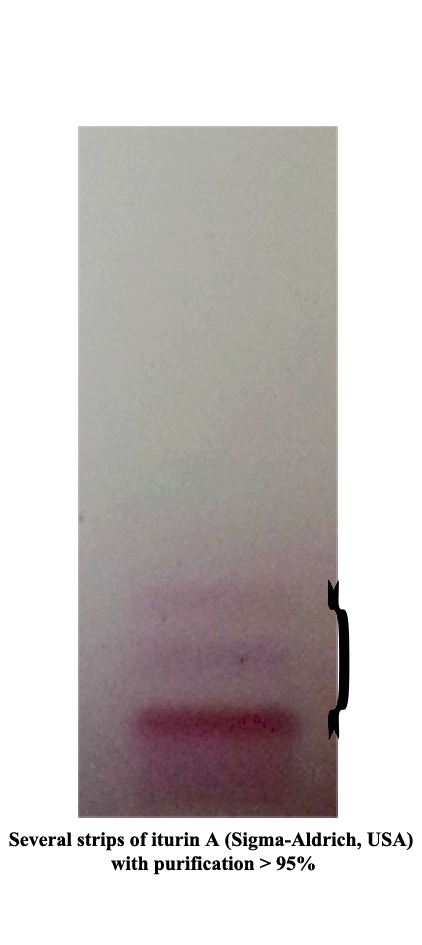

Supplement: Supplementary file 6 [file Image5.TIF]
